# Shoulder transcutaneous electric nerve stimulation decreases heart rate via potentiating vagal tone

**DOI:** 10.1038/s41598-021-98690-6

**Published:** 2021-09-27

**Authors:** Chun-Ming Hsieh, Wan-Chen Lin, Hsien-Yu Peng, Huang-Chung Chen, Yu-Cheng Ho, Chi-Jui Li, Xi-Guan Wu, Jen-Yi Chung, Shin-Da Lee, Tzer-Bin Lin

**Affiliations:** 1 Division of Physical Therapy, St. Paul’s Hospital, Taoyuan, Taiwan; 2 Department of Rehabilitation, St. Paul’s Hospital, Taoyuan, Taiwan; 3 Department of Physiology, School of Medicine, College of Medicine, Taipei Medical University, No. 250, Wu-Hsing Street, Taipei, 11031 Taiwan; 4 National Taiwan University Hospital, Taipei, Taiwan; 5 Department of Medicine, Mackay Medical College, New Taipei, Taiwan; 6 Department of Occupational Therapy, Asia University, Taichung, Taiwan; 7 Department of Physical Therapy, Graduate Institute of Rehabilitation Science, China Medical University, Taichung, Taiwan; 8 School of Rehabilitation Science, Shanghai University of Traditional Chinese Medicine, Shanghai, China; 9 Department of Bioinformatics and Medical Engineering, Asia University, Taichung, Taiwan; 10 Department of Biotechnology, Asia University, Taichung, Taiwan; 11 Cell Physiology and Molecular Image Research Center, Wan Fang Hospital, Taipei Medical University, Taipei, Taiwan

**Keywords:** Neuroscience, Physiology, Risk factors

## Abstract

By enhancing vagal activity, auricle transcutaneous electric nerve stimulation (TENS) is developed as a non-invasive therapy for heart failure. Nevertheless, though shoulder TENS used for treating adhesive capsulitis could affect vagal tone, its potential impact on heart functions remains unclear. In this study, electrocardiogram (ECG) and heart rate (HR) of subjects in response to sham, right-sided, or left-sided shoulder TENS (TENS-S, TENS-R, and TENS-L, respectively; 5 min) were recorded and analyzed. During the stimulation period, TENS-R constantly and TENS-L transiently decreased the HR of subjects; both TENS-R and TENS-L increased powers of the low- and high-frequency spectra. While TENS-R exhibiting no effect, TENS-L increased the ratio of low/high-frequency power spectrum indicating TENS-R decreased the HR through potentiating cardiac vagal tone. Collectively, these results suggest TENS could be an early and non-invasive therapy for heart failure patients before considering implant devices or devices are not feasible; moreover, therapists/physicians need to carefully monitor the potential adverse events during treatment for patient safety.

*Trial registration*: The study protocol was registered in ClinicalTrials.gov (NCT03982472; 11/06/2019).

## Introduction

The vagus nerve, a long meandering nerve bundle passing through the neck to the thorax and abdomen, extensively innervates and regulates the physiology of visceral organs including the heart^1^. Clinical studies demonstrate that biomimetic vagus nerve stimulation exhibits a pleiotropic protective effect in heart failure patients, as it improves ejection fraction of the ventricle^2^, grade of New York Heart Association classification^3^, baroreflex function^4^, and T-wave alternans^4^.

Transcutaneous electrical nerve stimulation (TENS) is a non-invasive technique in which a low-voltage current is delivered through surface electrodes to stimulate nerve fibers beneath the skin^5^. Shoulder TENS is a widely prescribed regiment for the patient suffering from adhesive capsulitis^5^; and in this case, the location of stimulation pads is very close to the base of the neck where the vagus nerve branches and enters the thorax^1^. For a very recent study have demonstrated auricle TENS modifies the heart rate (HR) of subjects by activating the vagus nerve^6^, we wonder if shoulder TENS used for treating capsulitis could also activate the vagus nerve to modify cardiac functions. Thereby, the impact of shoulder TENS on heart physiology was investigated by assaying its effects on the HR of subjects.

Because the vagal innervation of the heart is asymmetrical, i.e., the right vagus nerve has a more extensive synapse on the sinoatrial node^7^; and in heart failure patients, applying electrical impulses directly to the right vagus nerve results in a more pronounced HR decrement than to the left^8^, we thereby compared the changes of HR in response to TENS applied to the right and left shoulder. Moreover, for heart rate variability (HRV) is a recognized approach that non-invasively assays the autonomic regulation of HR^9^, we analyzed the HRV of subjects to clarify the possible neural mechanism underlying the TENS-induced HR change. To mimic clinical scenarios, a built-in waveform used for treating adhesive capsulitis in commercial TENS equipment was used in this study.

## Methods

### Ethical approval

This study complied with the Declaration of Helsinki, and was registered in ClinicalTrials.gov (NCT03982472 11/06/2019). All protocols were approved by the ethics committee of Taoyuan General Hospital, Ministry of Health and Welfare, Taoyuan, Taiwan.

### Study design

**Figure 1:**
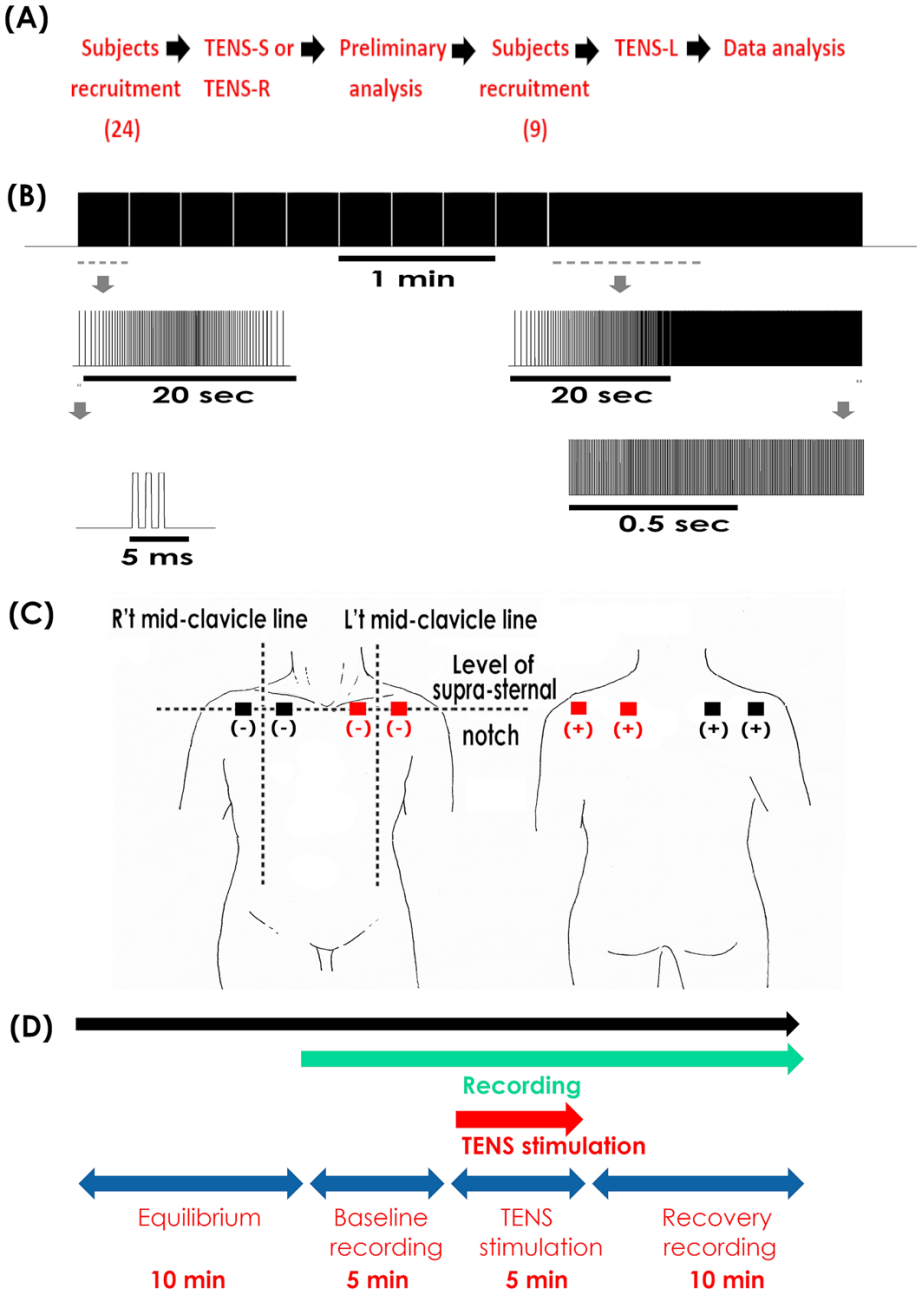
(**A**) The flow chart of participant recruitment and data analysis. Initially, 24 subjects were assigned to receive either the sham stimulation (TENS-S) or TENS on the right shoulder (TENS-R). After the preliminary analysis, 9 additional subjects were recruited and tested with TENS on the left shoulder (TENS-L). Data obtained from all experiments were then collectively analyzed. (**B**) The waveform of TENS. The upper trace is the waveform of TENS used in the current study. Waveform marked by the dash lines is amplified as the middle traces. Bottom left: each spike is a triple pulse with 1 ms pulse duration and each separated by a 1 ms interval. Bottom right: the frequency maintained at 200 Hz in the late period of stimulation. (**C**) The location of electrodes of TENS. Black blocks indicate the location of electrodes in TNES-S and TENS-R; red blocks indicate that in TENS-L. Stimulating electrodes ( −) were placed in the front of the should at the level of the suprasternal notch at about 2 finger-width medial and lateral from the mid-clavicle line; dispersive electrodes ( +) were placed at the back of the shoulder opposite to the stimulating electrodes. (**D**) The follow chart of TENS application. Before the stimulation, there was an equilibrium period of 10 min and then the recording started for 20 min. TENS was turned on after a 5 min baseline recording and lasted for 5 min. The recording stopped at 10 min after TENS offset.

The inclusion criteria of subjects were (1) less than 65, and (2) above 18 years old; and exclusion criteria were a history of (1) cardiovascular, (2) neurological, or (3) other medical (such as diabetes or inflammation) problems. Participants were recruited for one year, and the recruitment ended for there was enough data size. All subjects gave informed consent and were told that they are allowed to quit the test at any time or for any reason. If subjects displayed uncomfortable signs or harmful reactions (such as chemical burn) tests would be quitted. If interim analyses revealed any adverse reactions, this study would be ended. All the tests were conducted in the physical therapy department in St. Paul’s Hospital, Taoyuan, Taiwan. 33 subjects participated in this study; and initially, 24 subjects were randomly assigned to receive either the sham stimulation (TENS-S) or right-sided TENS (TENS-R) using a randomizer https://www.randomizer.org/. After analyzing preliminary data, 9 additional subjects were recruited to receive left-sided TENS (TENS-L) (Fig. 1A). Investigators were kept blind to the testing group until they prepared the TENS equipment. Though participants could distinguish the side and onset of TENS for the tingling sensation caused by simulation, the investigate would do his/her best to keep participants blind about their groups before, during, and after stimulation.

### ECG and HR recordings

Using a monitor lead, an electrocardiogram (ECG) was recorded through electrodes connected to a recording system (Biopac MP36, Biopac Systems, Santa Barbra, CA) with a sampling rate of 5000 samples/s. The ECG and the HR were calculated by a built-in rate meter. For TENS caused marked artifacts in ECG tracings, the HR was confirmed off-line by manual examinations.

### TENS stimulation

To mimicking clinical scenarios, a commercial TENS equipment (Dynaprog 528, Ever Prosperous Instruments, Taipei, Taiwan) was used in this study. A built-in waveform recommended for adhesive capsulitis in the user’s manual was used (triple pulses with 1 ms pulse durations separated by 1 ms intervals that ramps up and down between 2 and 10 Hz within 20 s for 3 min and then ramps up from 10 to 200 Hz within 20 s and kept at 200 Hz for 2 min; Fig. 1B). The current intensity was the maximal tolerable level below the pain threshold^5^. A pair of stimulating electrodes were placed in the front of the should at the level of the sternal notch at about 2 finger-width from the mid-clavicle line, and a pair of dispersive electrodes were done at the back of the shoulder opposite to stimulating electrodes (Fig. 1C)^5^. Participants kept a stationary supine position and were asked to refrain from moving during the recording period. Before the stimulation, there was an equilibrium period for at least 10 min, and then the recording started. TENS was turned on for 5 min after a 5 min baseline recording, and the recording continued until 10 min after the offset of TENS (Fig. 1D). Parameters of the sham stimulation were identical to the right-sided TENS excepting the equipment was left un-powered. No change was made to trial outcomes after the trial commenced.

### Spectrum analysis

For the stimulation frequency in this study (approximately 18–600 Hz) was far beyond the range of spectrum for analysis (0.04–0.4 Hz)^9,12^, the targeted spectrum can be decomposed separately and evaluated independently with minimal interference from the stimulating artifacts. Using a built-in software (BSL PRO 3.7, Biopac Systems), ECG signals were off-line transferred into a power spectrum using a nonparametric method of fast Fourier transformation. The direct current component was deleted and a Hamming window was used to attenuate signal leakage. A widely used frequency range, i.e., 0.04–0.15 Hz as low frequency and 0.15–0.40 Hz as high frequency, were used for analyses^9^.

### Statistical analysis

All data in this study were expressed as mean ± SEM. After checking the normality and variance of data, two-way ANOVAs were used to assess difference in values among testing groups (i.e., TENS-S, TENS-R, and TENS-L) and time points; and post hoc Student–Newman–Keuls tests were used to compare the means of groups when there was a significant difference between groups. Significance was set at *p* < 0.05.

## Results

### Database of subjects

Initially, 24 subjects were assigned to receive either the sham stimulation (TENS-S) or TENS on the right shoulder (TENS-R). After the preliminary analysis, 9 additional subjects were tested with TENS on the left shoulder (TENS-L). 1 of the subjects whose baseline HR was beyond the normal range (60–100 beats/min) did not complete the protocol. In 32 subjects included in the statistical analysis, 15 subjects were male (47%; 29.66 ± 2.95 years old) and 17 subjects were female (53%; 32.29 ± 2.34 years old). The TENS-S group included 10 subjects (male/female = 5/5; 28.30 ± 3.05 years old), the TENS-R group included 13 subjects (male/female = 7/6; 29.15 ± 2.64 years old), and the TENS-L group included 9 subjects (male/female = 3/6; 36.88 ± 3.77 years old). No significant difference in gender or age was observed between groups.

### TENS decreased HR in subjects

**Figure 2:**
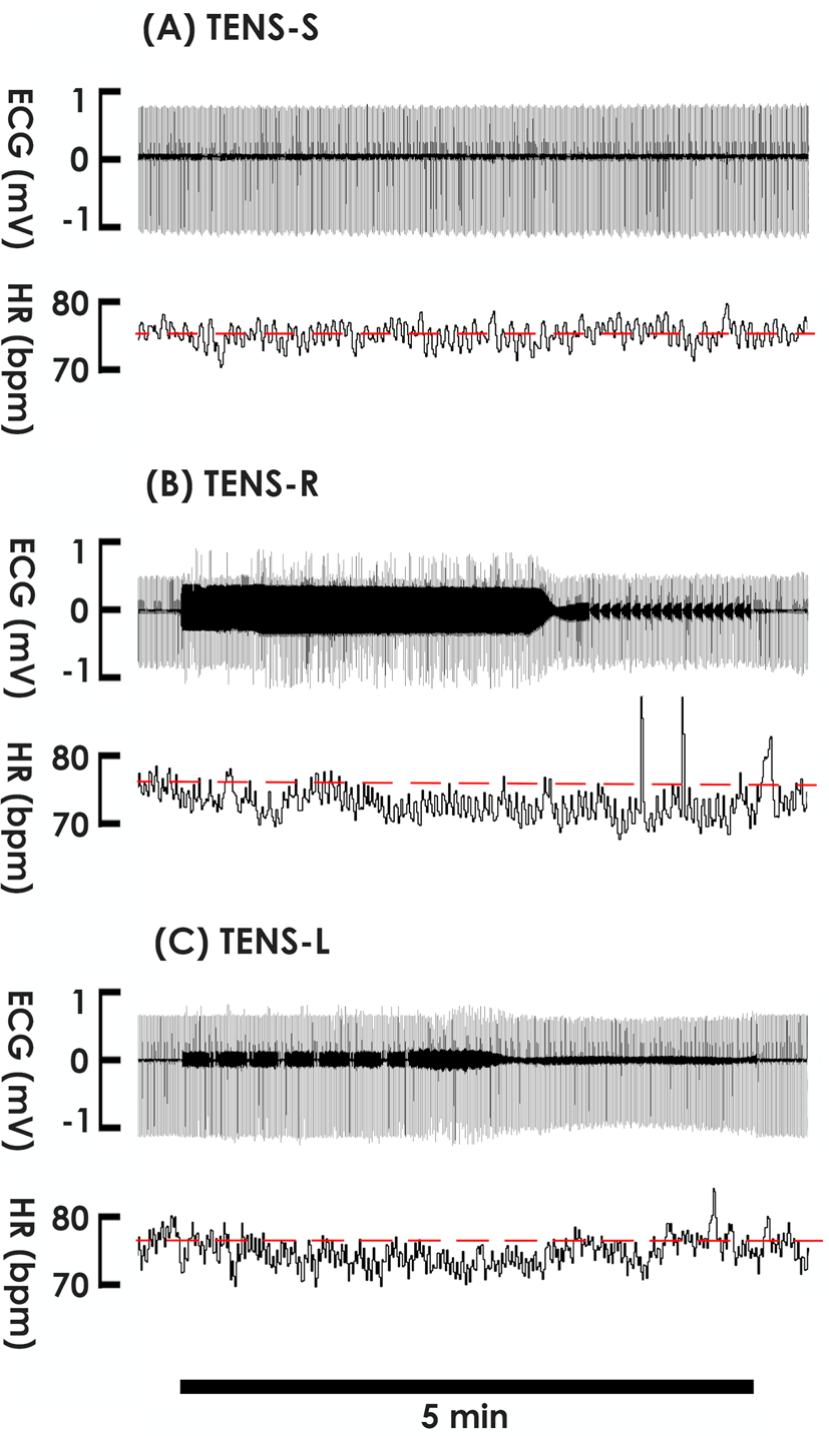
Electrocardiogram (ECG) and heart rate (HR) of subjects in response to (**A**) sham stimulation (TENS-S) and (**B**, **C**) TENS on the right and left shoulder (TENS-R and TENS-L, respectively) for 5 min. The black bar at the bottom indicates the stimulation period.

**Figure 3:**
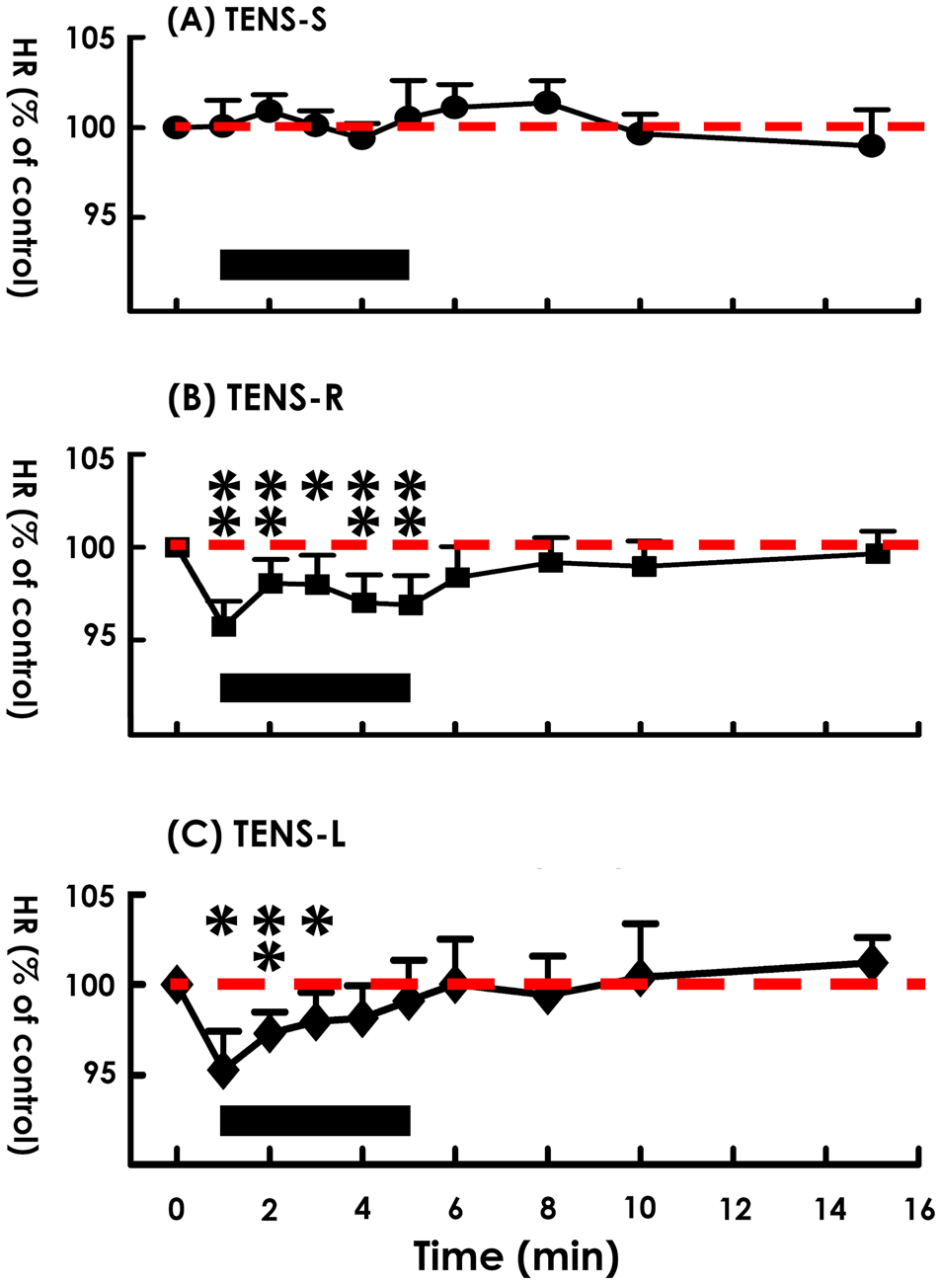
Mean heart rate (HR) of subjects in response to (**A**) sham stimulation (TENS-S) and (**B**, **C**) TENS on the right and left shoulder (TENS-R and TENS-L, respectively). The black bar at the bottom indicates the stimulation period. DF = 2, F = 5.31; *p* < 0.01 among groups; DF = 9, F = 1.94; *p* < 0.05 among time points; Two-way ANOVA. *p < 0.05; **p < 0.01 versus baseline control; post hoc.

**Figure 4:**
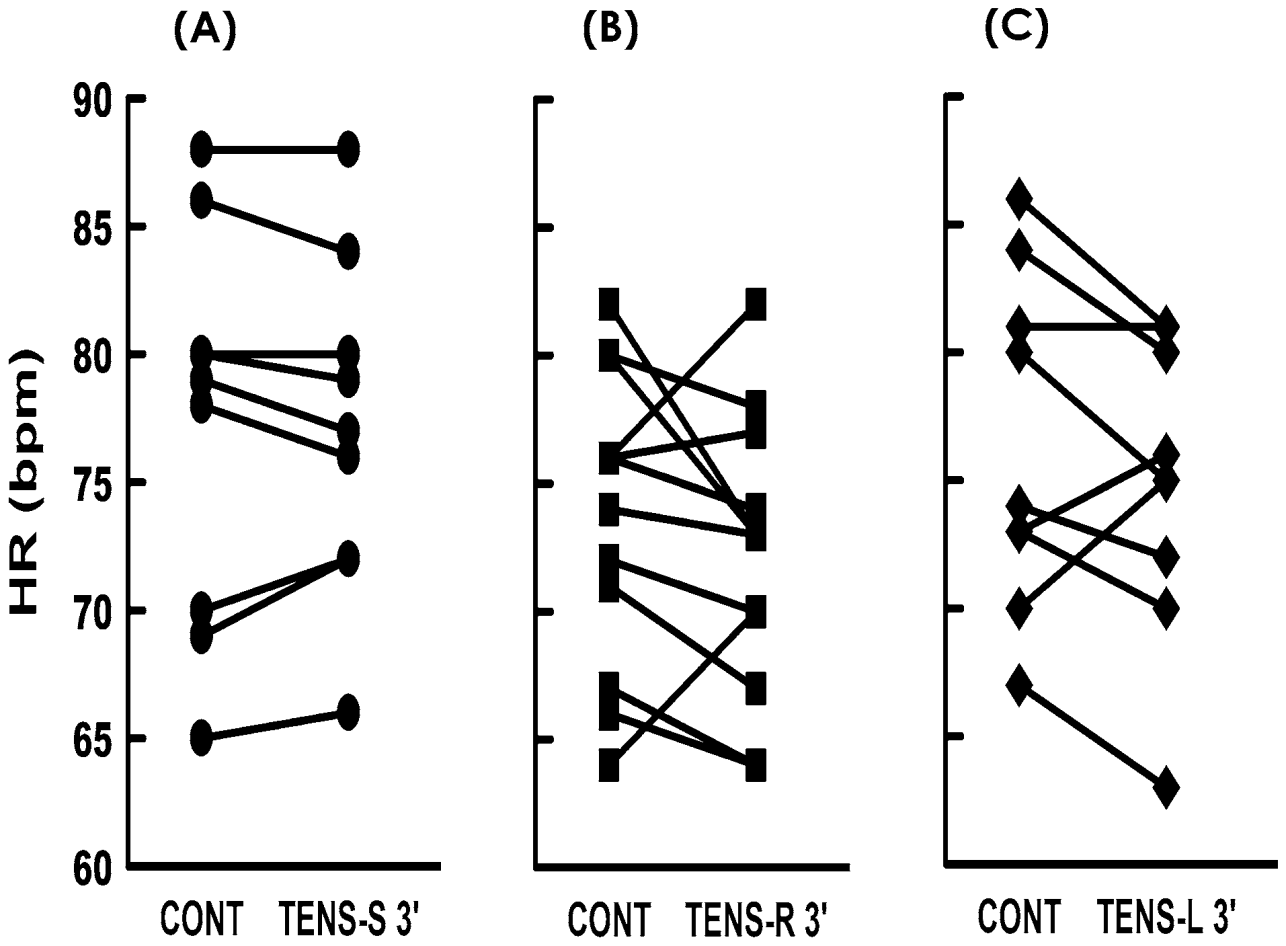
Individual heart rate (HR) of subjects at baseline control (CONT) and 3 min following (**A**) sham stimulation (TENS-S 3’) and (**B**, **C**) TENS at the right and left shoulder (TENS-R 3’ and TENS-L 3’, respectively). Mean HR of CONT and TENS-S/R/L 3’: (**A**) 77.30 ± 2.31 and 77.00 ± 1.98, (**B**) 73.84 ± 1.55 and 72.23 ± 1.49, and (**C**) 76.44 ± 2.18 and 74.77 ± 1.95 bpm, respectively.

The impact of TENS on cardiac rhythm was first investigated by applying TENS to the right shoulder of subjects. In contrast to TENS-S displayed no effect, (Fig. 2A), TENS-R slightly decreased the HR of subjects during the stimulation period (Fig. 2B) evidenced by it significantly decreased mean HRs at 1, 2, 3, 4, and 5 min following stimulation onset (Fig. 3B), while TENS-S failed to affect mean HRs at these time points compared with the baseline control (Fig. 3A). At the midpoint of the stimulation period (3 min), most TENS-R subjects (10 out of 13, 76%, Fig. 4B. Note: there were 2 overlapping data points) displayed a decreased HR compared to the baseline control; and 3 subjects exhibited an HR decrement more than 5% of baseline control. Quite different from TENS-R, in the TENS-S group, HR was decreased in 4 (40%) but was increased in 4 (40%) and remained unchanged in 2 (20%) of the 10 subjects (Fig. 4A Note: there were 2 overlapping data points); and no subject displayed an HR change more than 5% of baseline control.

### TENS altered autonomic tone in subjects

**Figure 5:**
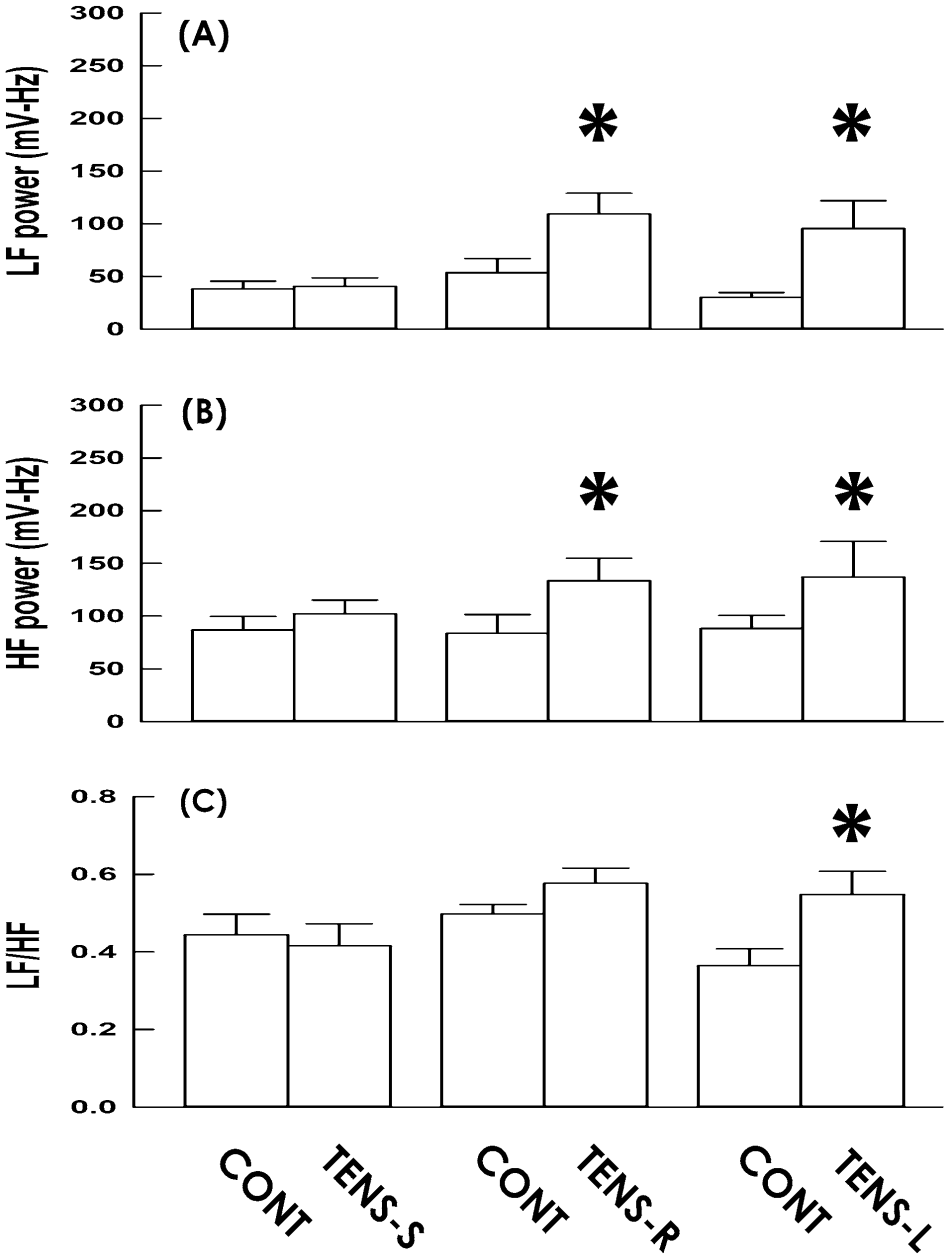
Spectrum analysis of heart rate variability. (**A**, **B**) Power of the low-frequency (LF) and high-frequency spectra as well as (**C**) Power ratio of LF to HF spectrum (LF/HF) at baseline control (CONT) and during the period of sham stimulation (TENS-S) and TENS on the right and left shoulder (TENS-R and TENS-L, respectively). (**A**–**C**) all DF = 2, F = 3.48, 3.30, and 3.19, respectively; all *p* < 0.05 among groups; all DF = 1, F = 21.63, 21.04, and 15.40, respectively; all *p* < 0.01 between treatment; Two-way ANOVA. *p < 0.05 versus baseline control; post hoc.

If the TENS-modified cardiac rhythm was accompanied by an altered autonomic tone of the heart was examined by comparing the HRV at baseline (5 min) and during stimulation (5 min). While TENS-S exhibited no effects, TENS-R significantly increased the power of the low-frequency (LF; an index of cardiac sympathetic and parasympathetic tones)^9^ and high-frequency (HF; an index of cardiac parasympathetic tone)^9^ spectra compared to the baseline control (Fig. 5A, B, respectively), suggesting TENS-R enhanced both sympathetic and parasympathetic activity of the heart. Moreover, TENS-R did not affect the LF/HF ratio (an index reflecting a predominance of cardiac sympathetic over parasympathetic tone)^9^, implying that TENS-R impacts cardiac vagal tone to a greater extent than sympathetic activity.

### Left-sided TENS decreased HR

To further clarify the role of vagal tone potentiation in the TENS-decelerated heart rhythm, we applied TENS to the left shoulder (TENS-L) of subjects. Although TENS-L also slightly decreased HR (Fig. 2C); nevertheless, in contrast to TENS-R constantly deceased the mean HR during the entire stimulation period, TENS-L transiently decreased the mean HR only at 1, 2, and 3 min; and the mean HR gradually recovery toward the baseline at 4 and 5 min following stimulation onset (Fig. 3C), indicating TENS-L and TENS-R coincidently but unequally decelerated heart rhythm. When compared with the baseline control, the HR was decreased in the majority (Fig. 4C; 6 out of 9 subjects, 66%), increased in some (2 subjects, 22%), and unaffected in 1 of TENS-L subjects at the midpoint of the stimulation period; and no subjects displayed an HR decrement more than 5% from the baseline. Comparable to TENS-R, TENS-L increased the power of the LF and HF spectra (Fig. 5C), indicating TENS-L also activated both cardiac sympathetic and parasympathetic outflow. Nevertheless, TENS-L significantly increased the LF/HF ratio, indicating this protocol activated a greater predominance of cardiac sympathetic over vagal tone.

## Discussion

### TENS decelerates heart rhythm via potentiating vagal tone

To assay the potential impact of TENS on the cardiac rhythm, we observed both TENS on the right and left shoulder regions rapidly but slightly decreased HR; and we suggested the decelerated rhythm is attributed to the TENS-potentiated vagal tone. Our proposal is based on the following lines of evidence. First, the position of stimulating electrodes in this study was very close to the anatomical location of the vagus nerve; and as its name indicated, TENS current is able to transcutaneously stimulate the nerve trunk beneath surface electrodes. This point is supported by a study showing TENS near the location of the cervical vagus nerve efficiently activates the vagus nerve in subjects without direct contact to the nerve trunk^10^.

Moreover, the vagus nerves asymmetrically regulate cardiac functions with the right-sided nerve more extensively innervates the sinoatrial node than the left-sided^7^. Compared with applying bioelectric stimulation directly to the left trunk of the vagus nerve, right-sided stimulation provokes a greater bradycardic effect in canines^11^. In healthy subjects stimulated with a transcutaneous unit^12^ and heart failure patients stimulated with an implanted device^8^, applying current to the right vagus nerve induces a more pronounced HR decrement than to the left. Analogously with these studies, our results demonstrated TENS-R and TENS-L with identical parameters synergistically but asymmetrically modified HR, i.e., TENS-R induced a sustained but TENS-L did a transient effect during the stimulation period; and TENS-R decreased HR in most of (76%) but TENS-L did in a majority of (66%) subjects. Collectively, such a synergistic but asymmetric HR decrement caused by the TENS-R and TENS-L further support for the involvement of vagal tone activation in the TENS-reduced HR.

Finally, HRV analyses demonstrated both TENS-R and TENS-L increased the spectrum power of the LF, a component that reflects the cardiac sympathetic and parasympathetic tone, as well as the HF, a marker of cardiac vagal activity^9^, indicating applying TENS current at the left and right shoulder both efficiently potentiated cardiac sympathetic and parasympathetic tone in subjects. Although to simultaneously enhance sympathetic and parasympathetic activity is physiologically unexpected, a preclinical study has demonstrated vagal stimulation of intact vagal nerves reflexively activates sympathetic outflow in dogs possible via the vagal-sympathetic reflex^13^. Interestingly, quite different from TENS-L increased the LF/HF ratio, an index representing a predominance of cardiac sympathetic over vagal activity^9^, TENS-R did not affect the LF/HF ratio; suggesting TENS-R provoked less vagal-sympathetic reflex activity than TENS-L did, and thereby results in a more pronounced HR decrement. Though the final proof needs future studies investigating subjects that are conditional on vagotomy or specific pharmacological antagonizing cardiac vagus nerve, results in this study collectively suggest the TENS-decelerated cardiac rhythm could be attributed to the potentiation of vagal tone. Not only analogously links the stimulation-triggered vagal tone to the TENS-induced HR decrement as shown in studies using earpiece TENS^6,11,17^, our data for the first time demonstrates a potential impact on cardiac rhythm could be induced by shoulder TENS, a regiment widely used in clinical practices.

### Potential clinical adverse effects and applications

Our findings have two implications; to the first, TENS on the shoulder region, particularly at the right side, could induce an adverse HR decrement. Even though our sample size was limited and TENS-R did not reduce HR in all participants, 10 of 13 subjects displayed HR decrease; and 3 of them exhibited a decrement of more than 5% of the baseline HR that is a level presumed to be an excessive HR alteration^11^. Despite studies are warranted to define the dose–response relationship between TENS and HR decrement, our results suggest clinicians need to be aware of the potential impact of TENS on cardiac rhythm when treating patients with adhesive capsulitis, particularly in those who have autonomic dysfunctions and/or arrhythmia. Our opinion is in agreement with studies indicating that avoiding excessive bradycardia is clinically important when developing vagal stimulation as a neural modulation of ventricular functions^14^, as it could be a safety issue in clinical scenarios^7^. In addition, a safe treatment protocol, including the location of electrode pads and the waveform/intensity of the applied current, merits further studies to provide a harmless therapy.

On the other hand, findings in this study possibly open up a novel application of TENS in physiotherapy practices as a means to noninvasively modify heart functions. Our proposal is based on that implanted vagal stimulating devices are approved by the US Food and Drug Administration for treating refractory epilepsy^15^ and resistant depression^16^; and though it causes side effects, including hoarseness, cough, and pain, implanted vagal stimulation devices effectively decrease HR during the stimulation period in heart failure patients^8^. Comparable to earpiece devices, a well-established technique that the sensory auricular branch of the vagus nerve is stimulated transcutaneously^17^, exhibits a marked HR deceleration in healthy subjects^6,11^ results in this study demonstrated that shoulder TENS also slightly but significantly decreased HR. Notably, quite different from an ear-stimulating TENS unit, which is designed for self-treatment daily and/or as necessary before onset of a seizure or paroxysmal supraventricular tachycardia, should TENS is a broadly used therapy in clinical practices where the safety of treatment sessions is under the supervision of a physician and/or therapist. We thereby suggest shoulder TENS is potential to be a supplement treatment for heart failure because lasting vagal stimulation exhibits a pleiotropic protective effect in heart failure patients^2–4^ and this protocol could offer an earlier non-invasive therapy before an implant device is considered or patients who are not feasible to an implanted stimulator.

### Possible neural machineries involved

Similar to experiments that acutely stimulate the vagus nerve^8^, in the current study, TENS-induced HR decrement appeared restrictively to the stimulation duration; and following TENS offset, HR recovered within a short period thereby lost therapeutic benefits. Yet, for lasting vagal stimulation exhibits protective effects in heart failure patients^2^, it suggesting long-term vagal stimulation could induce plastic changes in the autonomic nervous system regulating heart physiology^12^. Though candidate mechanisms, such as causing damage in the stellate ganglion^19^ and modifying cholinergic neurotransmission in nerve endings^10^ have been proposed, detailed machinery underlying the carryover effect of vagal stimulation is an interesting issue that needs to be further investigated.

Instead of regular square wave pulses delivered from a standard stimulator, or parameters of a constant pulse width (around 0.1–0.5 ms) and frequency (around 1–25 Hz) with a duty cycle of about 50% in the auricle TESN^19,20^, we applied subjects with a build-in waveform output from commercial equipment that stimulating frequency was ramp up and ramp down (2–10 and 10–200) with a duty cycle more than 90%. Though our protocol faithfully mimicked clinical scenarios in the physiotherapy department, further studies are warranted to elucidate if high stimulation frequency and/or duty cycle displays faster/higher therapeutic benefits. Moreover, stimulating subjects using square wave pulses, in which the amount of injected current can be assayed precisely, will clearly define the dose–response relationship between TENS and HR decrements.

### Controversy in the laterality of vagal stimulation

A study has recently demonstrated left-sided cervical vagal nerve stimulation caused stronger bradycardic, hypotensive, and tachypnea effects in anesthetized hypertensive rats than right-sided stimulation^21^. Whether species difference underlies such a discrepancy; or general anesthesia and/or pathological hypertension modify the laterality of cardiac vagal tone needs to be clarified. Notably, for several nerves and blood vessels that pass through the cervical region, applying current directly to such a narrow region is clinical contraindicated for it could reflexively induce laryngeal spasm and/or rapid fall in blood pressure^22^.

### Potential bias

In this study, we initially allocated participants into two groups; and participants were recruited into an additional group for the result of the preliminary analysis revealed TENS could activate vagal tone. Such an unusual recruitment protocol could give rise to selection bias that affects the results of this study. On the other hand, though we have tried our best to keep participants blind in their treatment before TENS stimulation, as it was clear that the TENS was applied to the right or the left shoulder, or if the power was not on for the sham group, the patient did know what treatment they received. Further studies ruling out the potential bias caused by inadequate blinding are needed to clarify the effects of TENS.

### Conclusion

Results in this study demonstrated a potential impact of TENS on autonomic tone regulating cardiac rhythm in clinical practices; and thereby not only suggested TENS as a potential supplement therapy for cardiac diseases but also reminded therapist/physician to carefully monitor the potential adverse events for patient safety.

## Data Availability

The datasets of the current study are available and can be provided upon reasonable requests from the corresponding author Tzer-Bin Lin (tblin2@gmail.com).
